# Enhanced electromechanical coupling of a nanomechanical resonator to coupled superconducting cavities

**DOI:** 10.1038/srep19065

**Published:** 2016-01-12

**Authors:** Peng-Bo Li, Hong-Rong Li, Fu-Li Li

**Affiliations:** 1 Institute of Quantum Optics and Quantum Information, Department of Applied Physics, Xi’an Jiaotong University, Xi’an 710049, China

## Abstract

We investigate the electromechanical coupling between a nanomechanical resonator and two parametrically coupled superconducting coplanar waveguide cavities that are driven by a two-mode squeezed microwave source. We show that, with the selective coupling of the resonator to the cavity Bogoliubov modes, the radiation-pressure type coupling can be greatly enhanced by several orders of magnitude, enabling the single photon strong coupling to be reached. This allows the investigation of a number of interesting phenomena such as photon blockade effects and the generation of nonclassical quantum states with electromechanical systems.

Cavity optomechanics^1^,^2^,^3^ and electromechanics^4^,^5^,^6^ are pretty promising for fundamental studies of large-scale quantum phenomena as well as appealing applications in quantum science and technology. Recent experimental progresses have demonstrated ground state cooling of the mechanical resonators^7^,^8^, coherent coupling between cavity and mechanical modes^9^,^10^,^11^, optomechanically induced transparency^12^,^13^, and the generation of squeezed light^14^,^15^. In despite of these remarkable advances, however, there is a serious hindrance in this exciting field, i.e., the radiation-pressure coupling is too weak to ensure dynamics of the system in the single photon strong coupling regime. Current experiments have mainly relied on strong optical driving, which enhances the coupling at the expense of making the effective interaction linear. As a result, this linear optomechanical interaction does not possess the ability of generating nonclassical states or give rise to true photon-photon interactions for implementing single-photon quantum processes.

To give a better understanding and fully exploit the regime of strong radiation pressure coupling, it is highly desirable to find an efficient method for realizing the strong nonlinear interaction between the vibrations and the electromagnetic field in a realistic setup. Such a regime is particularly important to test the fundamental theory of quantum physics and to explore potential applications of optomechanical or electromechanical devices to future quantum technology^16^,^17^,^18^,^19^,^20^,^21^,^22^,^23^,^24^,^25^,^26^,^27^,^28^,^29^,^30^. Some recent theoretical proposals for entering the strong coupling regime include the use of collective effects in arrays of mechanical oscillators^31^ and Kerr nonlinearity via the Josephson effect^32^,^33^, as well as the usage of an inductive coupling to a flux-dependent quantum circuit^34^.

Recently, a proposal using the squeezed optical cavity field to enhance the nonlinear coupling in an optomechanical system has been introduced^35^. In that proposal, the single-mode cavity field is squeezed by an optical *χ*^(2)^ nonlinear medium, while the broadband-squeezed vacuum is introduced to suppress the noise of the squeezed cavity mode. Though this protocol seems promising, it can not be straightforwardly applied to electromechanical systems. First, that proposal needs an optical nonlinear crystal possessing a large *χ*^(2)^ nonlinearity, which is quite demanding and technically challenging in the field of electromechanics with microwave frequencies. Secondly, in order to have implications in quantum information science, it is desirable to consider multi-cavities rather than a single cavity for the purpose of distributed quantum computation and quantum network^36^. However, the generalization of this model to the case of coupled optical cavities is still difficult. Fortunately, the above issues can be overcome by considering two coupled superconducting coplanar waveguide (CPW) cavities with the parametrical coupling form^37^,^38^.

In this work, we investigate an electromechanical system consisting of a nanomechanical resonator capacitively interacting with two parametrically coupled CPW cavities. We show that, when the cavities are driven by a spectrally broadband two-mode squeezed vacuum, the radiation-pressure type coupling of the resonator to the cavity Bogoliubov modes can be greatly enhanced by several orders of magnitude. By suitably tuning the system parameters such as the squeezing parameter of the driving source, the flux driving frequency and the parametric coupling strength, the single photon electromechanical coupling strength can be tailored such that it can exceed the cavity decay rate. This single photon strong coupling of electromechanical interactions allows the studies of single-photon quantum processes such as photon blockade and the production of nonclassical photon states harnessing the optomechanical nonlinearity. With currently available technology in cavity electromechanics, this proposal can be realistically implemented in experiments and provides an appealing platform for implementing quantum technologies.

## Results

### The setup

**Figure 1:**
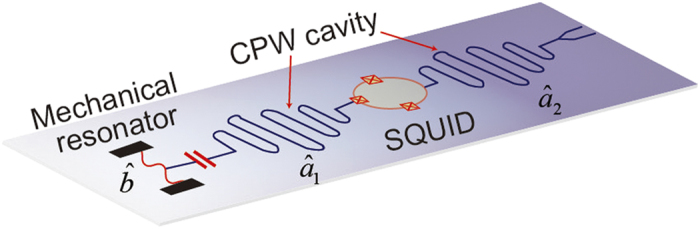
Schematic of the proposed electromechanical setup. A nanomechanical resonator capacitively couples to a superconducting CPW cavity, which is also coupled to another cavity with the form of parametric coupling. These cavities are driven by a spectrally broadband two-mode squeezed vacuum from the output of a non-degenerate Josephson parametric amplifier.

As shown in Fig. 1, we consider an electromechanical system consisting of a nanomechanical resonator capacitively coupled to a superconducting CPW cavity, which is also coupled to another auxiliary waveguide cavity by means of a superconducting quantum interference device (SQUID)^37^,^38^. The SQUID driven by external fluxes allows a fast modulation of the electrical boundary condition of the cavities and their interaction^37^,^38^. In a recent experiment, it has demonstrated a 3 D microwave superconducting cavity parametrically coupled to a transmission line cavity by a Josephson ring modulator^39^. The mechanical resonator couples to the cavity field via radiation-pressure coupling, while the two cavities interact with each other with the form of parametric coupling. In the frame rotating at half the flux driving frequency *ω*_d_, the system Hamiltonian reads


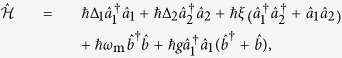


where 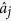 is the annihilation operator for the *j*th cavity mode with frequency *ω*_*j*_, Δ_*j*_ = *ω*_*j*_ − *ω*_d_/2, *ξ* the parametric coupling strength between the cavities, *ω*_m_ the mechanical vibration frequency with annihilation operator 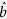, and *g* the radiation-pressure coupling strength between the resonator and the first cavity. The cavities are driven by an external source of two-mode squeezed microwave field, which can be produced with a non-degenerate Josephson parametric amplifier^40^,^41^,^42^,^43^. Assuming that the bandwidth of the squeezed microwave field is larger than the cavity damping rate *κ*, then the interaction between the cavity modes and the external squeezed field is described by^44^


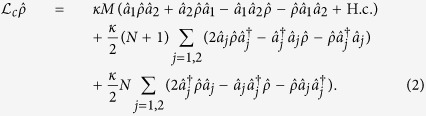


Here *M* and *N* are related to the statistics of the driving broadband two-mode squeezed field: *M* accounts for the intermode correlations, and *N* is the average photon number for both modes. For perfect two-mode squeezed vacuum, we have *M* = sinh *r*_0_ cosh *r*_0_ and *N* = sinh^2^ *r*_0_, with *r*_0_ the squeezing parameter. Therefore, the master equation describing the system dynamics reads


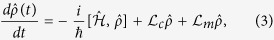


where


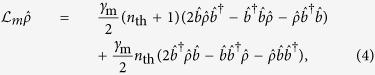


with *γ*_m_ the mechanical decay rate, and *n*_th_ the thermal phonon number of the mechanical mode.

### Enhanced electromechanical coupling via squeezed source driving

To get more insight into the system’s dynamics, it is convenient to introduce two delocalized cavity Bogoliubov modes^45^,^46^,^47^


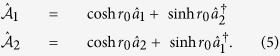


Using these cavity Bogoliubov modes, the cavity driven term can be rewritten as


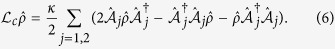


This means that for the cavity Bogoliubov modes, the dissipation caused by the system-bath coupling is just like that induced by a vacuum bath. Furthermore, if we choose 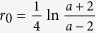, with 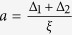, then through straightforward derivations, the system Hamiltonian can be written as


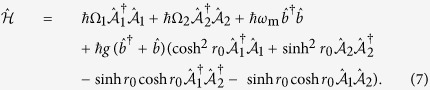


Here


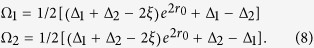


In the interaction picture with respect to


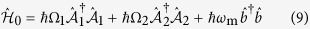


and under the condition Ω_1_ + Ω_2_ ≫ *g* sinh *r*_0_ cosh *r*_0_, *ω*_m_, this allows us to selectively activate interaction terms in the system dynamics as


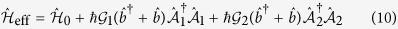


with 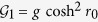 and 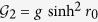. In the case of 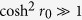, and 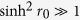, one has 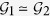. This Hamiltonian describes the electromechanical coupling between the resonator and the cavity Bogoliubov modes 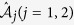 with the effective coupling strengths 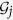. Together with the cavity dissipation described by (6) and mechanical decay, the system dynamics is just like that a mechanical resonator simultaneously couples to two photonic modes, with photon dissipation caused by a vacuum bath. In this case, the radiation-pressure coupling strength 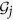 can be greatly enhanced. By suitably tuning the system parameters Δ_1_,Δ_2_, *ξ* and the driving source parameter *r*_0_, this coupling strength can be increased by several orders of magnitude to reach the strong-coupling regime, i.e., 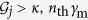. *This is the main result of this work*: by coupling the mechanical resonator to cavity Bogoliubov modes, and driving the cavities by a broadband two-mode squeezed vacuum, the radiation-pressure coupling can be greatly enhanced, even allowed to reach the strong coupling regime.

**Figure 2:**
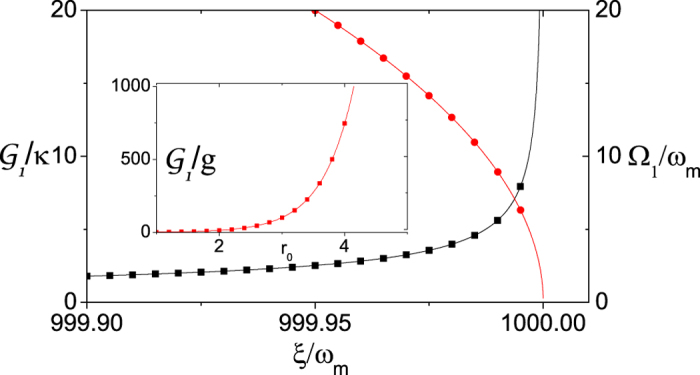
The radiation pressure coupling strength 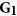, and the Bogoliubov mode frequency Ω_1_ versus the system parameter *ξ*. The relevant parameters are Δ_1_ + Δ_2_ = 2000*ω*_m_, *g* = 0.001*ω*_m_, and *κ* = 0.02*ω*_m_. In the inset 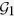 versus the squeezing parameter *r*_0_.

Figure 2 presents the calculated coupling strength 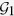 and the Bogoliubov mode frequency Ω_1_ as functions of the system parameter *ξ*. The results for 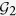 and Ω_2_ are similar to those for 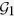 and Ω_1_ as presented in Fig. 2. As the parameter *ξ* approaches the critical value *ξ*_0_ = 0.5(Δ_1_ + Δ_2_), the coupling strength 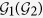 can get a very large value, while the Bogoliubov mode frequency Ω_1_(Ω_2_) tends to be very small. However, there is an optimal point at which both the coupling strength 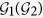 and the frequency Ω_1_(Ω_2_) can get a relatively large value, i.e., 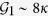 and Ω_1_ ~ 8*ω*_m_. The Bogoliubov mode frequencies Ω_1_ and Ω_2_ are controllable frequencies, which are determined by the driving frequency detunings Δ_1_, Δ_2_ and the parametric pumping strength *ξ*. They should have a relatively large value as compared to the parameters *g* sinh *r*_0_ cosh *r*_0_ and *ω*_m_. In this case we can ignore the parametric amplification terms for the Bogoliubov modes in Eq. (7) and obtain the standard electromechanical radiation-pressure interactions displayed in Eq. (10). This confirms that the single photon strong coupling can be reached with the chosen parameters.

We now consider whether the parameter regime is experimentally accessible with current experimental setups. From Fig. 2 we find that the scheme works well when *ξ* ~ 1000*ω*_m_. For nanomechanical resonators with frequencies from several kHz to several MHz, the parametrical coupling strength *ξ* between the cavities is on the order of hundreds of MHz. This coupling strength is well accessible with current circuit QED setups^39^. The value of *g* ~ 0.001*ω*_m_ is an order of magnitude lager than that achieved via mechanical resonators with frequencies of several MHz in current technology^5^. However, by the usage of mechanical resonators with low frequencies of hundreds of kHz, the value of *g* ~ 0.001*ω*_m_ is accessible in present experiments^48^. From the inset of this figure, we also note that this proposal requires a two-mode squeezing source with a very large squeezing parameter *r*_0_. Though this requirement is somewhat challenging, it is within reach of the state-of-the-art experiments. Recent experiments in two-mode squeezing of microwave radiation rely on the amplification of quantum noise by the Josephson Parametric Converter^40^,^41^,^42^,^43^. It has been demonstrated that the generated two-mode squeezing source can possess the highest gain of 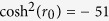 dB, which corresponds to a squeezing parameter *r*_0_ ~ 6.5. Therefore, this protocol can be realized with the state-of-the-art techniques of superconducting integrated circuits and electromechanical systems.

### Applications

The effective strong coupling offers great potential for single-photon manipulation and quantum states generation. For example, the photon blockade phenomenon existing in the strongly coupled optomechanical system can occur in this system. This is quantitatively characterized by the zero-delay second-order correlation function


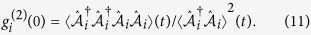


This quantity provides a direct experimental measure for nonclassical antibunching effects if 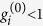 and for 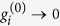 indicates a full photon blockade for the *i*th Bogoliubov mode.

We now discuss the feasibility of the measurement of the photon statistics in experiments. A challenge here is that the Bogoliubov modes are a linear combination of the two cavity modes, and are more difficult to be addressed separately. We assume the first cavity is weakly driven by a probe field with frequency *ω*_p_ and amplitude 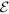. The Hamiltonian is given by


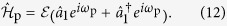


In the frame rotating at half the flux driving frequency *ω*_d_ and in the Bogoliubov mode representation, we have


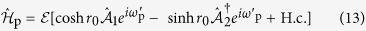


with 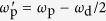. Under the condition 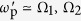, 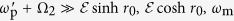, we can safely ignore the rapidly oscillating terms under the rotating-wave approximation


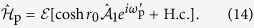


Therefore, with suitably choosing the probe parameters, one can selectively address the Bogoliubov modes on demand. The measurement of the photon statistics needs monitoring the signal coming out from the system and it is not known how the detailed measurement goes.

**Figure 3:**
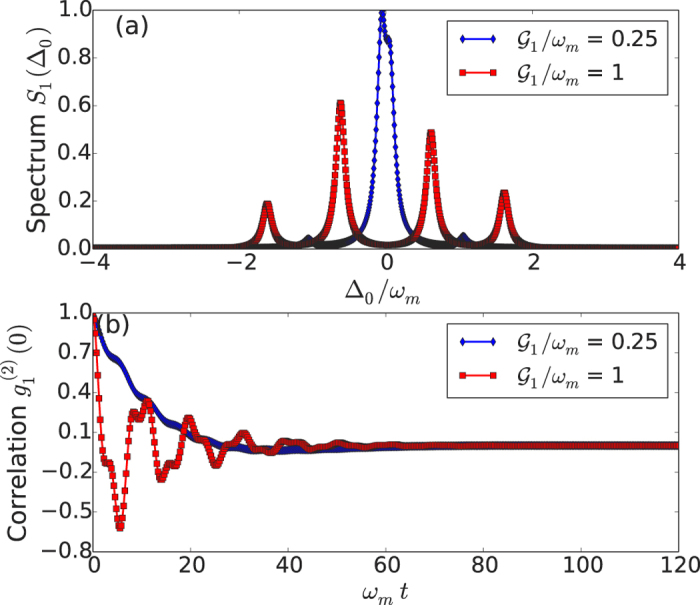
(**a**) Cavity excitation spectrum *S*_1_(Δ_0_) with different values of the coupling strength 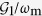 in the resolved sideband regime *κ* ~ 0.1*ω*_m_. (**b**) The second-order correlation function 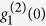 versus time. In both plots, *T* = 0 and *Q* = 10^3^.

In Fig. 3(a), we plot the excitation spectrum for the first Bogoliubov mode


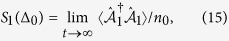


obtained by numerically solving Eq (3), as a function of the detuning 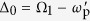 for different values of the coupling strength 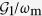, with 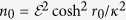. We observe in the resolved sideband regime *κ* ≪ *ω*_m_, a redshift of the zero phonon line (ZPL) towards 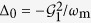 and the appearance of additional resonances at multiples of the mechanical frequency. These peaks result from phonon-assisted excitation processes, providing a clear indication for single-photon strong coupling optomechanics. In Fig. 3(b) we present numerical results for the second-order correlation function 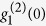 versus time through solving the master equation (3) with the transformed Hamiltonian (7). It is clear that the steady state value for 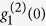 approaches zero for the first Bogoliubov mode, which is a strong signature of photon blockade.

## Discussion and Conclusion

In this work, we focus on implementing the idea using two parametrically coupled superconducting cavities in an electromechanical system. The usage of two cavities rather than one is particularly appealing when one considers applying this proposal to quantum information processing such as distributed quantum computation and quantum network. This proposal can also apply to a single microwave cavity capacitively coupled to a mechanical resonator. In this case, it will need a single-mode squeezed field to drive the cavity, and a superconducting qubit in the cavity to produce the desired nonlinearity for the cavity mode^49^.

To conclude, we have proposed an efficient method for enhancing the radiation-pressure type coupling in an electromechanical system. We have shown that, by driving the cavities with a two-mode squeezed microwave source and selectively coupling the resonator to the cavity Bogoliubov modes, the effective interaction strengths between the mechanical resonator and the cavities can be enhanced into the single-photon strong-coupling regime. This allows the investigation of a number of interesting phenomena such as photon blockade effects and the generation of quantum states with currently available technology in the field of cavity electromechanics.

## Additional Information

**How to cite this article**: Li, P.-B. *et al.* Enhanced electromechanical coupling of a nanomechanical resonator to coupled superconducting cavities. *Sci. Rep.* **6**, 19065; doi: 10.1038/srep19065 (2016).
